# Combination of Ethanolic Extract of Citrus aurantifolia Peels with Doxorubicin Modulate Cell Cycle and Increase Apoptosis Induction on MCF-7 Cells 

**Published:** 2014

**Authors:** Anugerah Budipratama Adina, Fina Aryani Goenadi, Franciscus Feby Handoko, Dwi Ana Nawangsari, Adam Hermawan, Riris Istighfari Jenie, Edy Meiyanto

**Affiliations:** a *Cancer Chemoprevention Research Center Faculty of Pharmacy, University of Gadjah Mada Sekip Utara II, Yogyakarta, 55281, Indonesia. *

**Keywords:** *Citrus aurantifolia *peels, Doxorubicin, MCF-7, Cell cycle, Apoptosis

## Abstract

New approach of breast cancer therapy is developed toward combination therapy with agents that have a specific molecular target. Our previous study showed that *Citrus aurantifolia *lime peels ethanolic extract (CPE) increased the sensitivity of MCF-7 cells againts doxorubicin. This study aims to observe the mechanism of combination CPE and doxorubicin in cell cycle modulation and apoptosis on MCF-7 cells. The assays were performed in the study were cell cycle assay, apoptosis induction, and immunocytochemistry of MCF-7 cells.The effect on the modulation of cell cycle and apoptosis were observed by flowcytometry assay in both single dose of CPE and its combination with Doxorubicin. Cell cycle distribution were observed with flowcytometer FACS-Calibur and its data was analyzed by Cell Quest program. Apoptotic induction in MCF-7 cells was examined using acrydine orange-ethidium bromide (AO-EtBr) double staining. Immunocytochemistry assay was done to observe the expression of apoptotic regulation protein p53 and Bcl-2.

The result showed that CPE 6 μg/mL induced apoptosis and cell accumulation at G1 phase, while CPE 15 μg/mL induced apoptosis and cell accumulation at G2/M phase. The combination of doxorubicin 200 nM with CPE 6 μg/mL increased apoptosis induction than their single treatment, and cell accumulation at G2/M phase. Evidence of apoptosis and protein expression of p53 and Bcl-2 indicated that both single applications and combinations of CPE and doxorubicin are able to increase apoptotic bodies of MCF-7 cells by increasing the proteins expression. This result suggested that CPE could perform as co-chemotherapeutic agent with doxorubicin on breast cancer cells.

## Introduction

The use of doxorubicin as chemotherapeutic agent causes various problems such as drug resistance and toxic effect on normal tissue which main pressing the immune system and heart toxicity (1, 2). Chemoresistance of cancer cells leads to cell heterogeneity and failure to apoptosis (3). Failure of apoptosis cause cell growth was not restrained (4). Combination of chemotherapeutics and chemopreventive agent with target cell cycle modulation and apoptosis was conducted to increase efficacy of chemotherapeutics (5,6). 

Research revealed that the cancer cells have a lower capacity to perform the apoptosis due to p53 mutation and overexpression of Bcl-2 (7, 8). The capability of agent in increasing of p53 and decreasing of Bcl-2 expression on MCF-7 cells is important to increase the sensitivity against resistance of cancer cells. Therefore combinatorial treatment between chemotherapeutics and chemopreventive agent targeted to p53 and Bcl-2 is interesting issue to be explored. 

*Citrus aurantiifolia *is potential chemopreventive agent. Our previous study showed that CPE increased the sensitivity of MCF-7 breast cancer cell againts doxorubicin (9). Combinational treatment of CPE with doxorubicin in MCF-7 cells showed that the combination is able to give the synergistic effect (CI < 1) on with an optimum concentration of CPE-doxorubicin 1_/__8_ IC_50_-1_/__2_ IC_50_ (6 μg/mL of CPE and 200 nM of doxorubicin) (9). Other study also mentioned anticarcinogenic activity, apoptosis induction and antiproliferative of CPE on *in-vivo *breast cancer model induced by DMBA (10, 11). The aim of this research is to determine the mechanism of combination of CPE and doxorubicin in cell cycle modulation and apoptosis mediated by p53 and Bcl-2 expression. The results of this research are expected to become the basis of developing of combination therapy using the natural products that is *C. aurantifolia*.

## Experimental

*Materials*

*Citrus aurantiifolia *lime (harvested in Banyuwangi, East Java and identified by a botanist at Department of Pharmaceutical Biology, Universitas Gadjah Mada) peels (CPE) was extracted by using 70 % ethanol and concentrated by using freeze dryer to obtain concentrated extract. Doxorubicin was obtained from Ebewe. A DMSO (Sigma Aldrich Chemie GmBH, Steinheim, Germany) solution of CPE was used for *in-vitro *experiment by diluting appropriate consentration. Doxorubicin was diluted directly in culture medium. The final DMSO concentration was set at less then 1 %.

*Cells*

MCF-7 cells were cultured in *Dulbecco’s Modified Eagle’s Medium *(DMEM) containing *Fetal Bovine Serum *(FBS) (Gibco)10% (v/v) and penicillin-streptomycin 1 % (v/v) (Gibco). This cell lines were kindly provided by Prof. Masashi Kawaichi (Nara Institute of Science and Technology, Nara, Japan). 

*Flowcytometry assay*

MCF-7 cells with a density 5.10^5^ cells/well were transferred into 6-well plate and then incubated in 37 °C incubator (5% CO_2_) until the cells return to normal conditions. Cells were treated by sample solution and incubated for 48 hours. At the end of the incubation time, all the cells both adherent and floating are harvested using trypsin-EDTA 0.25%. Harvested cells were transferred into tube and centrifugated. Pellet cells in tube then were washed by cold PBS and and resuspended in PBS containing Propidium Iodide (PI) (Sigma Aldrich, GmbH) (40 μg/mL), RNAse (100 μg/mL) (Gibco) and TritonX-100 (Sigma Aldrich, GmbH) at 37 °C for 10 min. The samples were then analysed using FACS flowcytometer. Percentage of cells in each stage of cell cycle (sub G1, G1, S and G2/M phases) were calculated using Cell Quest program.

*Apoptosis examination*

MCF-7 cells with a density 5 x 10^4^ cells/well, were seeded on coverslips (Nunc) in 24-well plates (Iwaki) and incubated in 37 ºC incubator (5% CO_2_) until confluent. After that, cells were incubated with samples for 15 hours. Furthermore, the medium was taken and the plate containing the cells was washed with PBS. Coverslips containing cells were moved into object-glass and added by l0 μL 1X working solution acrydine orange (AO) (Sigma Aldrich)-etidium bromide (Et-Br) (Sigma Aldrich) double staining. after 5 minutes, cover slip immediately observed under the flouresense microscope (Zeiss MC 80). Viable cells had green fluoresense and apoptotic cells had orange fluoresense.

*Imunocytochemistry assay*

MCF-7 cells (5 x 10^4^ cells/well) were seeded on coverslips in 24-well plates (Iwaki) and incubated in 37 °C incubator (5% CO_2_) until confluent. Then, cells were incubated with samples for 15 h. Culture medium were removed and cells were washed in PBS. Cells were fixed with cold methanol for 10 minutes and washed by PBS. Then, H_2_O_2_ as blocking solution were added on the fixed cells for 10 minutes, removed, and normal mouse serum was added for 10 minutes, removed, and incubated with monoclonal antibody anti-p53 (Dako) and anti-Bcl-2 (Dako) at 4 °C over night. Then, cells were washed with PBS and incubated with biotinylated universal secondary antibody for 10 minutes, removed, and washed with PBS. Cells were incubated with streptavidin-peroxidase complex reagent for 10 minutes, removed, and washed with PBS. Cells were stained with substrate solution Diamino Benzydine (DAB) (Sigma Aldrich, GmbH) for 10 minutes, removed, and wash with aquadest. Cells were stained with Mayer Haematoxylin for 3 minutes, removed, and washed with aquadest. Coverslips were moved into object-glass and fixed with ethanol and xylol and added with mounting media, then covered by new coverslips. Protein expression observed by light microscope (Nikon YS100). Cells that express a particular protein will provide the color brown, while the cells that that does not give a specific protein will provide color blue.

## Results and Discussion

The study was aimed to evaluate the mechanism of CPE as a co-chemotherapy on doxorubicin treatment on MCF-7 cells. Our previous study showed that the ethanolic extract of *C. aurantifolia *(CPE) and doxorubicin are able to prevent the growth of MCF-7 cells with IC_50_ values of 59 μg/mL and 328 nM, respectively (9). This present study performed mechanism of the combination on modulation of cell cycle and apoptosis. 

**Figure 1:**
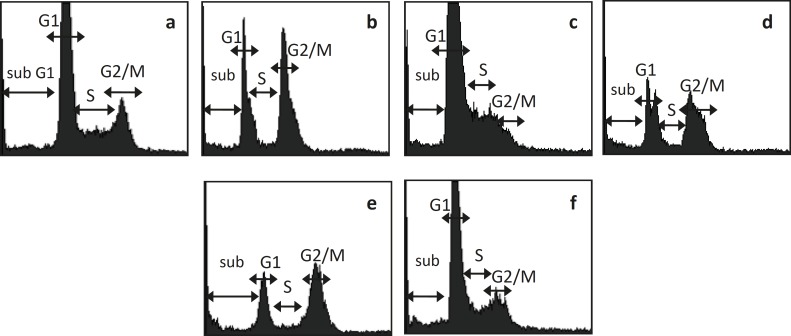
MCF-7 cell cycle analysis after treatment of Doxo, CPE, and their combination. MCF-7 cell were treated by Doxo, CPE, and their combination for 48 hours and stained by PI reagent before analyzed by flowcytometer. (a) control, (b) doxorubicin 200 nM, (c) CPE 6 μg/mL (d) CPE 15 μg/mL, (e) doxorubicin 200 nM-CPE 6 μg/mL, (f) doxorubicin200 nM-CPE 15 μg/mL.

Synergistic effect of combination between CPE and doxorubicin could be occured via cell cycle modulation (Table 1 and Figure 1). Cell cycle analysis of MCF-7 breast cancer cell lines showed that single treatment of Doxorubicin 200 nM induced G2/M arrest. Single treatment of CPE 6 μg/mL induced G1 arrest, while in higher dose (15 μg/mL) induced G2/M arrest. Combination of doxorubicin 200 nM-CPE 6 μg/mL induced cell accumulation in G2/M phase and apoptosis. Combination of doxorubicin 200 nM-CPE 15 μg/mL induced cell accumulation in G1 phase and apoptosis compared to control and doxorubicin single treatment. The increasing of CPE concentration in combination did not show increasing of apoptosis induction. The higher dose of CPE in combination, the lower of apoptosis induction occured, but it is still higher than apoptosis in doxorubicin single treatment.

**Table 1:** MCF-7 Cells distribution after treatment of CPE, doxorubicin, and their combination for 48 hours

**Treatment**	**Concentration** **(IC**_50_ **)**	**% of Cell in Phase**				
		**Sub G**_1_ **(apoptosis)**	**G**_1_	**S**	**G**_2_ **/M**
Control	-	7.02	66.05	10.61	14.31	
Doxorubicin	200 nM	10.64	31.74	4.31	47.52	
CPE	6 μg/mL	9.04	60.84	18.58	7.77	
CPE	15 μg/mL	18.14	32.22	4.77	40.40	
Doxo-CPE	200 nM-6 μg/mL	25.28	22.00	5.65	41.87	
Doxo-CPE	200 nM-15 μg/mL	18.40	52.42	9.64	15.10	

Cell growth retardation is possible through the cell cycle regulatory mechanisms. Single treatment of doxorubicin induced G2/M arrest because of inhibition of topoisomerase II(12). Single treatment of CPE in lower dose induced cell accumulation in G1 phase, it is probably because of the effect citrus flavonoid. Most of citrus flavonoid citrus flavonoid induced G1 arrest. Some of citrus flavonoids *e.g. *Tangeretin (13,14), naringin (15), tangeretin and nobiletin (16,17) and hesperetin (18) induced G1 arrest. Naringenin and hesperidin induced G1 arrest in MCF-7 cells (19). While single treatment of CPE in higher dose induced G2/M accumulation. It is probably because of the effect of other flavonoid like genistein that induced G/2M arrest by up regulating Cyclin B expression, but the mechanism the mechanism need to be explored further. 

Combination of doxorubicin and lower dose of CPE induced G2/M arrest and apoptosis induction compared to their single treatment. Previous study reported that p53 upregulating p21 resulted in downregulation of Cyclin B1/ Cdk1 and leads to G2/M phase arrest (20)**. **Combination of of doxorubicin and higher dose of CPE induced G1 arrest and apoptosis induction compared to their single treatment. Increasing dose of CPE induced decreasing of apoptosis, and increasing cell accumulation on G1 phase. The mechanism probably due to p53 decreasing WAFI/Cipi(20), but the exact mechanism need to be explored details. 

**Figure 2:**
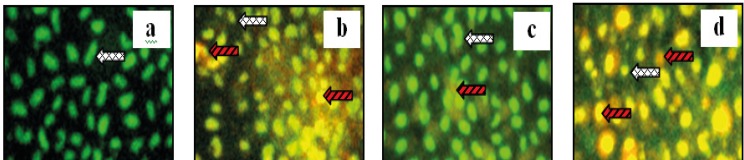
Apoptosis examination of combination of doxorubicin with CPE in MCF-7 cell line**. **MCF-7 cells with a density 5 x 10^4 ^cells/ well, were seeded on coverslips in 24-well plates until confluent. Than, plate was incubated with samples for 15 hours. Futhermore, double staining was done by AO-EtBr. (a) control, (b) Doxorubicin 200 nM, (c) CPE 6 μg/mL, and (d). Doxorubicin 200 nM- CPE 6 μg/ mL. Viable cell, dead cell with red fluorosense

Apoptosis examination of MCF-7 cells was performed by using CPE and doxorubicin, both a single application or a combination of both. This examination was to see the apoptosis of MCF-7 cells after treatment. Results of this research showed that the optimum combination of doxorubicin 200 nM-CPE 6 μg/mL induced apoptosis compared to the control cells, doxorubicin and CPE in single application (Figure 2). 

**Figure 3:**
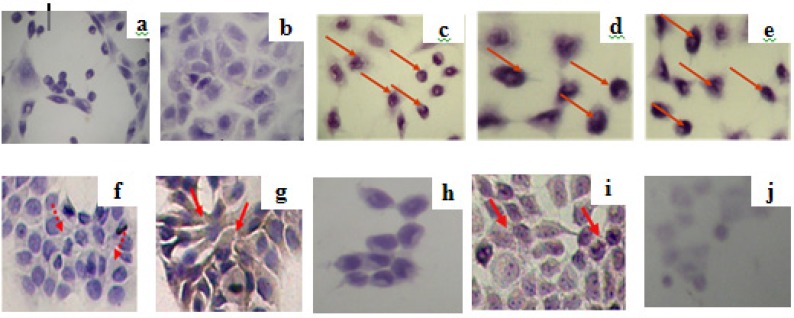
Imunocytochemistry assay of expression of apoptosis regulator proteins p53 (a-e) and Bcl-2 (f-j) by using combination of ethanolic CPE with doxorubicin in MCF-7 cell line. MCF-7 cells (5 x 10^4^ cells/well) were seeded on coverslips in 24-well plates until confluent. Than, plate was incubated with samples for 15 hours. Futhermore, imunocytochemistry was done. Protein expression observed by light microscope; (a,f) control without antibody; (b,g) control with antibodi, (c,h) CPE, (d,i) doxorubicin 200 nM, dan (e,j) doxorubicin 200 nM-CPE 6 μg/mL. Protein expression was pointed by red arrow

The observation of expression of apoptosis regulator proteins p53 and Bcl-2 was conducted in MCF-7 cells by using CPE and doxorubicin, both a single application or in combination of both. Imunocytochemistry assay with p53 antibody showed the expression of p53 of either single application of CPE or a combination of both (Figure 3a-e). Results of this research also shows that the optimum combination of doxorubicin-extract suppressed the expression of Bcl-2, therefore it’s strengthen that mechanism of apoptosis induction on MCF-7 cells through p53 activation pathways (Figure 3f-j). Upregulation of p53 on combination doxorubicin and CPE high dose probably correlated to G1 arrest, showed by flowcytometry. Probably combination of CPE and doxorubicin increased p21 and leads to G1 arrest, but the exact mechanism need to be explored details. 

Expression of p53 shown in this study is able to activate proapoptotic factors (Bax, Bak, Bcl- Xs, and Bad) and antiapoptotic (Bcl-2, BclXL, and Bcl-W). Citrus flavonoid isolated from *Citrus aurantiium *induced apoptosis on Human Gastric Cancer AGS Cells by upregulating Bax and downregulating Bcl-XL (21)**. **Another study reported that hesperidin, a citrus flavonoid, also induced p53 expression and apoptosis on NALM-6 cells (22)**. **Thus, it is posibly that apoptosis induction on combination of CPE and doxorubicin occurs through the same pathway, but we need to know the exact mechanism by observing p53 upstream protein expression. 

Single treatment of doxorubicin upregulated Bcl-2 on MCF-7 cells, while single treatment of CPE downregulated Bcl-2. Combination of CPE and doxorubicin downregulated Bcl-2 higher than CPE single treatment. Doxorubicin induced Bcl-2 expression via PI3K/Akt and activation of NFkB (23, 24). Citrus flavonoid isolated from *Citrus aurantium *inhibited PI3K/Akt pathway (25)**, **thus probably citrus flavonoid from *Citrus aurantiifolia *also performed the same mechanism, but needs to be clarified further. Hesperidin also performed inhibition of NFkB activation on NALM-6 cells (22), thus probably citrus flavonoids also act in the same mechanism, but we need to study further. 

A variety of research including this research to strengthen the presumption that the synergistic effect of ethanolic extract of *C. aurantifolia *was mediated by cell cycle modulation and apoptosis, so that doxorubicin treatment is more efficiently in the MCF-7 breast cancer cells. However, further research in specific molecular mechanism in breast cancer therapy needs to explore further. 

## Conclusion

Based on these results, ethanolic extract of *C. aurantifolia *is able to increase the sensitivity of MCF-7 cells to doxorubicin by cell cycle modulation and apoptosis induction. 
